# Massive Childhood Lead Poisoning: The Price of Nigerian Gold

**DOI:** 10.1289/ehp.120-a165a

**Published:** 2012-04-01

**Authors:** Adrian Burton

**Affiliations:** Adrian Burton is a biologist living in Spain who also writes regularly for *The Lancet Oncology*, *The Lancet Neurology*, and *Frontiers in Ecology and the Environment*.

Childhood lead poisoning on a scale unheard of for decades has been detected in rural northwestern Nigeria [*EHP* 120(4):601–607; Dooyema et al.]. The culprit: lead in gold ore processed using artisanal techniques. Chelation therapy for hundreds of children, soil replacement, and an education campaign to discourage processing ore inside homes may now have radically reduced child mortality in the hardest-hit villages, but the long-term effect of lead poisoning on the surviving children remains to be seen.

The outbreak surfaced in the spring of 2010 when health professionals noticed abnormally high rates of child illness and death among young children in 4 villages of Zamfara State. Blood tests on 8 children returned blood lead levels (BLLs) of 168–370 mg/dL, at least 16 times the level of concern set by the U.S. Centers for Disease Control and Prevention (CDC). The Nigerian authorities quickly assembled an international team to identify the source of the exposure and to respond, focusing on the 2 worst-affected villages.

Blood samples were collected from 59% of children under age 5. Of these, 97% had BLLs of at least 45 mg/dL, the threshold at which the CDC recommends chelation therapy. The BLLs of 85% surpassed the portable sampling devices’ maximum detection limit of 65 mg/dL.

**Figure:**
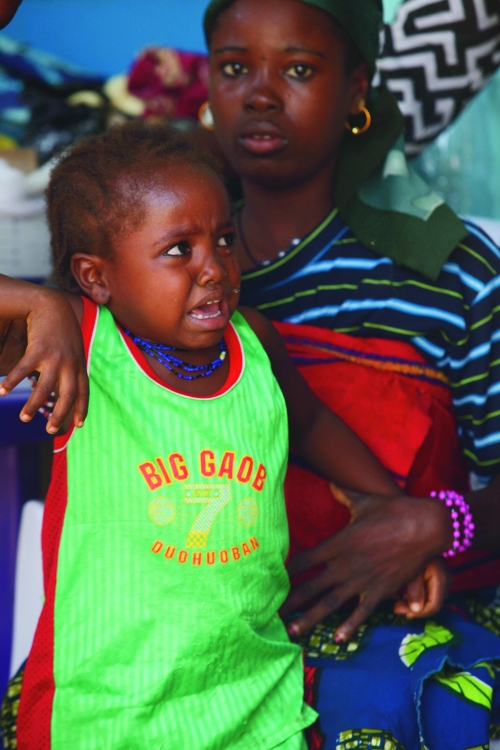
A child awaits treatment for lead poisoning in 2010. Nearly all children tested in the worst-affected villages had blood lead levels high enough to warrant chelation therapy. APN Photo/Sunday Alamba

A survey of the villagers revealed that 25% of all children under age 5 had died in the previous year, most of them in the half-year before the study. This translates to a mortality rate of 255/1,000 live births, compared with a national average of 157/1,000. The problem was the lead-contaminated gold ore being processed in many of the family compounds. Two-thirds of these families had started the activity within the last year.

Soil samples were collected from nearly all the family compounds where processing occurred, with 85% showing heavy lead contamination. The worst reached 250 times the U.S. Environmental Protection Agency safety limit of 400 ppm for play areas. Similarly, water lead concentrations far exceeded U.S. recommendations.

Not every child’s blood could be tested, and a lack of medical data for the deceased meant their deaths could not be definitively linked to lead poisoning. Further, the locally recruited survey staff had limited training in administering questionnaires and collecting environmental samples, which may have affected the results. Nonetheless, the evidence clearly suggests these villages were hit by lead poisoning due to artisanal processing of contaminated gold ore.

